# A Novel Oncogenic and Drug-Sensitive KIF5B-NTRK1 Fusion in Lung Adenocarcinoma

**DOI:** 10.3390/curroncol31110489

**Published:** 2024-10-24

**Authors:** Hui Li, Huicong Liu, Lisha Xiao, Huabin Gao, Huiting Wei, Anjia Han, Gengpeng Lin

**Affiliations:** 1 Department of Pathology, The First Affiliated Hospital, Sun Yat-sen University, Guangzhou 510080, China; lihui225@mail.sysu.edu.cn (H.L.); weiht3@mail.sysu.edu.cn (H.W.); 2 Department of Pulmonary and Critical Care Medicine, The First Affiliated Hospital of Sun Yat-sen University, Institute of Pulmonary Diseases, Sun Yat-sen University, Guangzhou 510080, China; liuhc7@mail2.sysu.edu.cn (H.L.);

**Keywords:** KIF5B-NTRK1, lung adenocarcinoma, entrectinib, signaling pathways, case report

## Abstract

We present a case of a lung adenocarcinoma patient harboring a novel kinesin family member 5B (*KIF5B*)-*NTRK1* gene fusion that responds well to entrectinib. Moreover, *KIF5B*-*NTRK1* gene chimera has been shown to be an oncogene, activating both the MAPK and PI3K/AKT signaling pathways. The biopsy sample was analyzed using various methods such as hematoxylin–eosin staining (HE), immunohistochemistry (IHC), fluorescence in situ hybridization (FISH), and next-generation sequencing (NGS) based on a 1267-gene panel. Additionally, human lung adenocarcinoma cell lines A549 and H1755 were used to obtain a stable expression of chimera gene products. The cell proliferation was confirmed using CCK8 and adhesion-dependent colony formation assay. Cell invasion was confirmed using the transwell invasion assay. The protein levels of the MAPK and PI3K/AKT signaling pathways were assessed using Western blotting. The patient, a 66-year-old Chinese male, was diagnosed with adenocarcinoma (stage IVB) located in the upper lobe of the left lung. NGS analysis identified a novel *KIF5B*-*NTRK1* fusion gene, which was further confirmed by FISH and IHC analyses. As a first-line therapy, entrectinib was administered to the patient at a dose of 600 mg once daily, resulting in a partial response. The patient’s progression-free survival (PFS) has now been more than 12 months, and no serious toxicities have been observed so far. Furthermore, stable KIF5B-NTRK1-expressing cells were generated and the experimental results demonstrate enhanced proliferation abilities, along with increased levels of proteins involved in the MAPK and PI3K/AKT signaling pathways. Our study reports a novel *KIF5B*-*NTRK1* genetic rearrangement that supports favorable responses to entrectinib. Moreover, in vitro experiments showed that the fusion gene could exert oncogenic properties by activating the MAPK and PI3K/AKT signaling pathways. To summarize, our findings broaden the spectrum of *NTRK* gene fusions in the context of lung adenocarcinoma.

## 1. Introduction

The family of neurotrophic tyrosine receptor kinase (NTRK) genes encodes for three tropomyosin receptor kinases (TRKs) (TrkA, TrkB, and TrkC proteins) [1]. Each of these TRK proteins consists of a transmembrane region, an intracellular kinase domain, and an extracellular domain. The intracellular domain contains five tyrosine (Tyr) residues at catalytically active sites, with three Tyr residues located in the activation loop and the other two Tyr residues on either side of the kinase domain, acting as phosphorylation-dependent docking sites for ligands and several other interacting cytoplasmic adaptors, including SHC-transforming protein (SHC), fibroblast growth factor receptor substrate 2 (FRS2), and phospholipase Cɣ-1 (PLCɣ-1) [2]. TRK proteins play pivotal roles in the regulation of maturation and differentiation of sympathetic and motor neurons, supporting the development of the central (CNS) and peripheral nervous systems (PNS) [3].

Previous studies have identified that there is a chimeric oncogene involving the tropomyosin 3 (*TPM3*) gene and a specific genomic locus of *NTRK1* in colorectal cancer (CRC) patients [4,5]. The identification of the *TPM3-NTRK1* gene fusion triggered several other studies to identify and characterize gene chimeras in cancers. In 1998, Knezevich et al. discovered the recurrent chimeric *NTRK3-ETV6* gene in 70% of patients with infantile fibrosarcoma (IFS) [6]. Notably, two other studies found the same *NTRK3-ETV6* fusion gene in cellular variants of congenital mesoblastic nephroma (CMN) [7,8]. Furthermore, Frattini et al. analyzed 185 tissue samples of glioblastoma multiforme (GBM), revealing two distinct gene chimeras by in-frame fusions between *NTRK1* and *NFASC* or *BCAN* [9]. *NTRK* gene fusions have also been reported in papillary thyroid carcinoma (PTC) [10], intrahepatic cholangiocarcinomas [11], breast cancer [12], and acute lymphoblastic leukemia (ALL) [13]. To date, over 80 different partners of gene fusions involving *NTRK* have been identified in cancers.

The frequency of *NTRK* gene rearrangements in non-small-cell lung carcinoma (NSCLC) is relatively low, accounting for approximately 0.2% [14]. Up to now, *NTRK1* gene fusion accounted for the largest proportion, with approximately 21 different *NTRK* fusion partners reported in NSCLC [15]. MPRIP-NTRK1 and CD74-NTRK1 fusion rearrangement in NSCLC, first described in 2013, has been shown to accelerate cell proliferation and tumor formation in nude mice [16]. Here, we report a novel *KIF5B-NTRK1*-fused chimeric in NSCLC and uncover its biological function in vitro. Interestingly, this chimeric gene product promoted cell proliferation and adhesion-dependent colony formation; however, the fusion protein did not affect the invasive property of NSCLC cells. The downstream of TRK signaling includes the MAPK, PI3K/AKT, and PLCγ pathways [17]. *MPRIP-NTRK1* and *CD74-NTRK1* fusion genes are shown to increase the phosphorylation levels of ERK1/2 and AKT in HEK293T cells [16]. Consistently, we demonstrate that *KFI5B-NTRK1* promotes the proliferation ability of NSCLCs via activating both the ERK/MEK and PI3K/AKT pathways.

Nowadays, two targeted agents are available for patients harboring NTRK gene fusions: larotrectinib and entrectinib. Both of them were designed to block the ATP binding site of the TRK family of receptors to exert antitumor activity [18,19]. Following a combined review of three clinical trials examining the activity in patients with locally advanced or metastatic NTRK fusion-positive solid tumors, the FDA awarded expedited clearance in 2018 and 2019, respectively [20,21]. Recently, updated results of the larotrectinib efficacy analysis showed the objective response rate was 69% (95% CI, 63–75%) and median progression-free survival was 29.4 months (95% CI, 19.3–34.3 months) [22], while the objective response rate of entrectinib was 57% (95% CI, 43.2–70.8%) and the median duration of response was 10 months (95% CI, 7.1–not evaluated) [21]. We also previously reported a novel *NOTCH2-NTRK1* rearranged chimera in an osimertinib-treated lung adenocarcinoma patient, which was effective for larotrectinib [23]. In this report, the patient received entrectinib and was followed for 12 months. He is currently in good health, with no distant metastasis, and the tumor has shrunk significantly.

## 2. Methods

### 2.1. Cell Lines and Reagents

Human lung adenocarcinoma cell lines A549 and H1755 were obtained from the Chinese National Infrastructure of Cell Line Resource, and the cells were cultured in RPMI-1640 medium containing 10% fetal bovine serum (FBS) and incubated at 37 °C with 5% CO_2_ in a humidified incubator. Antibodies against CK7 (#OV-TL12/30) and NapsinA (#MX015) were purchased from MXB Biotechnologies, China. Anti-TTF-1 (#SPT24), Anti-M-CEA (#12-140-10), and Anti-pan-TRK (#RM423) were purchased from ZSGB Biotechnologies, China. Antibodies against total AKT (#4691), phosphoAKT (Ser473) (#4060), total ERK (#4695), phosphoERK (Thr202/Tyr204) (#4370), total MEK (#9122), phosphoMEK1/2 (Ser217/221) (#9154), HA(C29F4) (#3724), and β-actin (13E5) (#4970) protein antigens, along with a secondary goat anti-rabbit IgG antibody (#7074) were obtained from Cell Signaling Tech. Anti-GAPDH (#47724) antibody was purchased from Santa Cruz Biotech. The Cell Counting Kit-8 detection kit (#C0037) and blasticidin (#ST018) were purchased from Beyotime Biotech, China. Entrectinib (#S7998) was purchased from Selleck Chemicals LLC, Houston, TX, USA.

### 2.2. Next-Generation Sequencing (NGS) and Data Processing

DNA was extracted and quantified from formalin-fixed paraffin-embedded (FFPE) tumor tissue and peripheral blood samples using the GeneRead DNA FFPE Kit (QIAGEN, Hilden, Germany) and Mag-Bind Blood & Tissue DNA HDQ 96 Kit (OMEGA Bioservices, Norcross, GA, USA), respectively. The purified DNA was examined using the Qubit dsDNA HS Assay Kit (Thermo Fisher Scientific, Waltham, MA, USA) for quality. A customized next-generation sequencing panel targeting exons of 1267 genes (YuceBio Technology Co., Ltd., Shenzhen, China) was used to prepare sequencing libraries, and the DNBSEQ-T7RS was used for sequencing with 100 bp paired-end reads. The mean depth of coverage was 1590× for tumor and 599× for matched blood control. Low-quality results with a N rate beyond 10% were filtered out using SOAPnuke (version 1.5.6). The clean results were aligned with the human reference genome hg19 using the Burrows–Wheeler Alignment tool (BWA, version 0.7.12) with BWA-MEM algorithms. Alignment data conversion, sorting, and indexing were carried out using SAMtools (version 1.3). Duplicates were marked with SAMBLASTER (version 0.1.22) to reduce biases. Delly software was used to identify structural variants (SVs) and annotate them. To remove false-positive SV calls, a random forest model was used.

RNA was extracted and quantified from FFPE tumor tissue using the RNeasy FFPE Kit (#73504, QIAGEN, Hilden, Germany). The purified RNA was checked for quality using the Qubit RNA HS Assay Kit (Thermo Fisher Scientific, Waltham, MA, USA). A 96-rxn Solid Tumor Fusion RNA-Cap Panel (BOKE, Beijing, China) was used to prepare sequencing libraries, and the DNBSEQ-T7RS was used for sequencing with 100 bp paired-end reads. The mean depth of coverage was 17,407×. The raw RNA-seq data were processed using STAR v2.5.3a, and gene fusions were detected using STAR fusion.

### 2.3. Fluorescence In Situ Hybridization (FISH) Assay

The *NTRK1* gene rearrangement was detected by a dual-color break-apart probe in FISH. The target gene was tagged with a spectrum green label at the 3′-end and a spectrum red label at the 5′-end. Samples were pre-treated by a Vysis Paraffin kit (#08N43-03, Abbott Molecular, Abbott Park, IL, USA) before hybridization. The samples were *NTRK1* gene rearrangement-positive if only a 3′ signal or a longer separation (>1 signal diameter) between the 5′ and 3′ signals was detected in at least 15% of the population in the tumor cells.

### 2.4. Immunohistochemistry (IHC)

FFPE tissue sections were stained with anti-CK7, anti-NapsinA, anti-TTF-1, anti-M-CEA, and anti-pan-TRK antibodies. Detection was performed in a Ventana Benchmark Autostainer (Roche, Basel, Switzerland).

### 2.5. Stable Cell Line Generation

The *KFI5B-NTRK1* fusion gene containing *KIF5B* exons 1–24 and *NTRK1* exons 12–19 was synthesized by GENEWIZ and subcloned into pLenti-MCS-P2A lentiviral expression plasmid, with an HA-Flag tag. The lentivirus particles were generated in HEK293T cells with packaging plasmid-pMD2.G and psPAX2. For stably expressing KFI5B-NTRK1 cell line generation, the A549 and H1755 cells were infected by lentivirus particles for 48 h and selected using blasticidin for 10 days.

### 2.6. Polymerase Chain Reaction (PCR)

The *KFI5B-NTRK1* cDNA was synthesized using PrimeScript™ II 1st Strand cDNA Synthesis Kit (#6210A, Takara, Dalian, China, TaKaRa Bio) and the following primer pairs: forward: 5′-TCGTGATCGCAAACGC-3′ and reverse: 5′-TGAGCACGATGTCCCG-3′. The reaction conditions were as follows: 95 °C for 10 min, 40 cycles of sequential 95 °C for 15 sec, 55 °C for 15 sec, and 60 °C for 60 sec. A 2% agarose gel electrophoresis was used to separate the PCR products, and ethidium bromide was used to visualize the band.

### 2.7. Cell Proliferation Assay

A549 and H1755 cells were seeded in 96-well plates, 2 × 10^3^ per well, and cell proliferation was measured according to the manufacturer’s instructions for the CCK8 kit (#B34304, Bimake, Shanghai, China). The absorbance values in triplicate of each well were recorded daily for four days.

### 2.8. Adhesion-Dependent Colony Formation Assay

A549 and H1755 cells were seeded in 6-well plates with 200 cells per well and incubated for 2 weeks. The cells were later washed twice with PBS, fixed using 4% paraformaldehyde, and stained with 0.5% crystal violet for 15 min. Using a microscope, the number of colonies in 10 randomly selected view fields was counted, and the average number of colonies was calculated. The experiment was performed independently three times.

### 2.9. Transwell Invasion Assay

The transwell chamber (Millipore, Darmstadt, Germany) was used to conduct the invasion assay. First, 50 µL of matrix glue was placed in the upper chamber and A549 and H1755 cells were seeded in the same chamber containing serum-free medium (10^5^ cells). The lower chamber was filled with RPMI-1640 medium supplemented with 20% FBS. After 48 h, the chamber was collected and the cells were fixed with pre-cooled 4% paraformaldehyde for 30 min. They were then stained with 0.1% crystal violet for 20 min. The membrane of the cells was removed and scanned for statistics. For each membrane, five random field counts were performed at 40× magnification. Three independent experiments were performed, and the data are presented as mean ± standard deviation (SD).

### 2.10. Western Blotting (WB)

After 48 h incubation, A549 and H1755 cells were collected and lysed in RIPA lysis buffer for 30 min at 4 °C and protein concentration was determined using the BCA kit (Thermo-Fisher, Waltham, MA, USA). A total of 30 µg protein per sample was separated by 10% SDS-PAGE and transferred onto the PVDF membrane. The membrane was blocked with 5% non-fat skimmed milk in 1 × TBST for 2 h at room temperature. The membrane was then incubated with primary antibodies for total AKT (1:1000), phosphoAKT (Ser473) (1:1000), total ERK (1:1000), phosphoERK (Thr202/Tyr204) (1:1000), total MEK (1:1000), phosphoMEK1/2 (Ser217/221) (1:1000), HA (1:1000), GAPDH (1:2000), and β-actin (1:1000) at 4 °C overnight. Then, a secondary goat anti-rabbit IgG (1:4000) antibody-conjugated horseradish peroxidase was applied to the membrane at room temperature for 1 h. Protein bands were imaged using an ECL Plus Western blot detection system (Sinsage Tech, Beijing, China). Densitometric measurement of each band was performed by ImageJ v1.6 (NIH, Bethesda, MD, USA).

### 2.11. Statistical Analysis

GraphPad Prism v8 and SPSS v25.0 were used for all statistical analyses. Student’s *t*-test (two-tailed, unpaired) was employed to compare cellular proliferation, colony formation, and invasion properties across different treatment and control groups. The data are expressed as mean ± SEM. A *p*-value of less than 0.05 was considered statistically significant.

## 3. Results

### 3.1. Case Description

**Figure 1:**
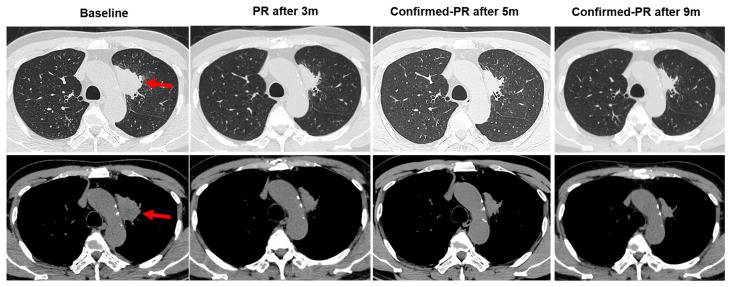
Changes in primary lesions on chest CT images pre- and post-entrectinib therapy. The chest computed tomography (CT) scan images showing the location of tumor lesions found in the left upper lung (red arrows). After taking entrectinib, 600 mg/day, orally for 3 months, the tumor showed a decrease in size; oral administration of entrectinib for 5 months indicated the tumor continued to shrink in size; oral administration of entrectinib for 9 months showed that the tumor was continuously reduced in size. PR: partial response.

**Figure 2:**
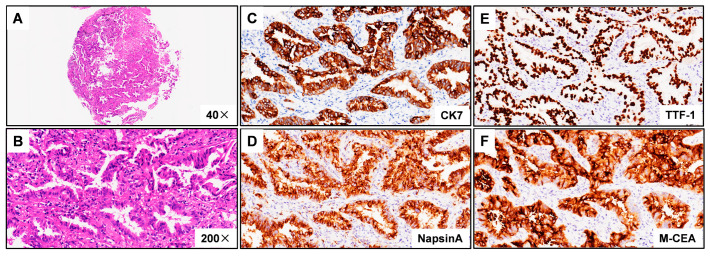
Hematoxylin–eosin (HE) staining and immunohistochemistry (IHC) images from patient harboring KIF5B-NTRK1 demonstrating lung adenocarcinoma. (**A**) Discovery of the KIF5B-NTRK1 fusions by DNA-based NGS testing. The breakpoints on KIF5B and NTRK1 are located on chr10:32305041 and chr1:156845146. (**B**) Identification of the KIF5B-NTRK1 fusions at the RNA level. (**C**) CK7 IHC staining demonstrating strong cytoplasm staining in tumor cells (×200). (**D**) NapsinA IHC staining demonstrating strong cytoplasm staining in tumor cells (×200). (**E**) TTF-1 IHC staining demonstrating strong nuclear staining in tumor cells (×200). (**F**) M-CEA IHC staining demonstrating strong cytoplasm staining in tumor cells (×200). Representative images are shown.

**Figure 3:**
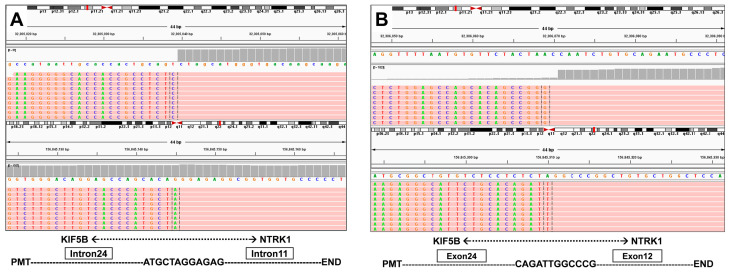
(**A**) Discovery of the KIF5B-NTRK1 fusions by DNA-based NGS testing. The breakpoints on KIF5B and NTRK1 are located on chr10:32305041 and chr1:156845146. (**B**) Identification of the KIF5B-NTRK1 fusions at the RNA level.

**Figure 4:**
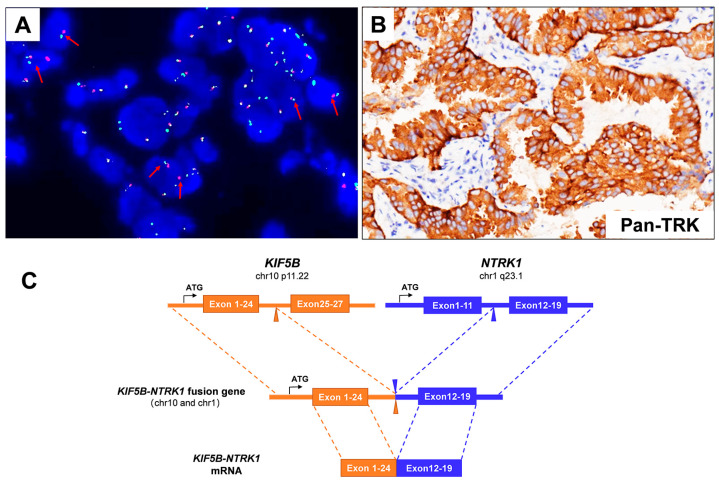
(**A**) The Vysis NTRK1 Break Apart FISH Probe Kit successfully identified the presence of NTRK1 fusion in the patient’s specimen, displaying a split signal (either green or red) at both the 5′ and 3′ ends of the NTRK1 gene (red arrows). (**B**) IHC staining indicated a strong Pan-TRK protein expression (×200). (**C**) A schematic map of the novel fusion gene, which was generated by the fusion of KIF5B (exons 1–24) with NTRK1 (exons 12–19).

A 66-year-old male Chinese patient, who had been smoking for more than 40 years, complained of coughing and chest pain. In October 2022, he was admitted to a local hospital. The patient had no history of coronary heart disease, hypertension, tuberculosis, diabetes, or any other infectious diseases. He had previously undergone a thyroidectomy for goiter in 2007, and his thyroid function remained normal after the operation. Following a physical examination and a series of auxiliary examinations, a computed tomography (CT) scan revealed a mass measuring 49 × 30 mm in the patient’s left upper lung (Figure 1). Additionally, multiple metastatic lesions were discovered in the brain and bones. A fiberoptic bronchoscope-guided biopsy of the lung tumor indicated adenocarcinoma (Figure 2), and he was diagnosed with stage IVB non-small-cell lung cancer (T2bN2M1c). To discover potential therapeutic regimens, an NGS-based profiling of 1267 genes was performed using biopsy tissues. A rare but novel KIF5B-NTRK1 rearrangement was discovered with an allele frequency of 10.25%, and it was identified at the RNA level (Figure 3A,B). The breakpoints on genes KIF5B and NTRK1 were located on chr10:32305041 and chr1:156845146, respectively. The result was confirmed by FISH and IHC approaches (Figure 4A,B). The chimeric gene contained exons 1–24 of KIF5B and exons 12–19 of NTRK1 genetic loci (Figure 4C). In addition, other co-mutations, including frameshift mutation in phosphatase and tensin homolog (PTEN) and copy number amplification of the genes FGF4 and MDM2, were also detected in this patient. No EGFR mutations or anaplastic lymphoma kinase (ALK) gene rearrangements were found. Thus, the patient received entrectinib (600 mg. PO. QD) as the first-line treatment in December 2022. The patient’s clinical symptoms improved dramatically after 3 months, and the shrinkage of primary lesions was significant (Figure 1). The first efficacy evaluation of the drug was determined by the partial response (PR), according to the Response Evaluation Criteria In Solid Tumors (RECIST), edition 1.1. So far, the patient has shown 12 months of progression-free survival (PFS), without any serious adverse events.

### 3.2. KIF5B-NTRK1 Promotes Proliferation and Adhesion-Dependent Colony Formation but Not Invasion

**Figure 5:**
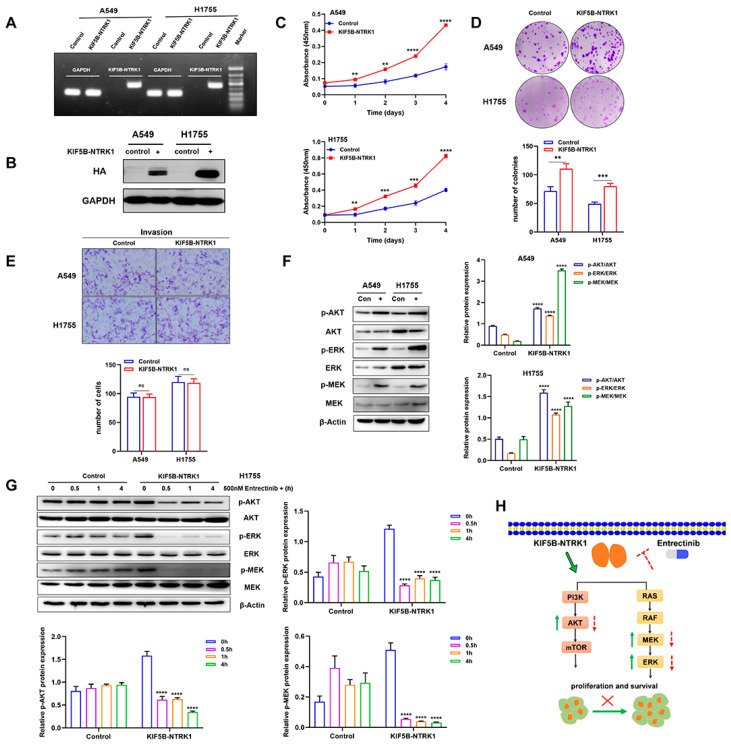
KIF5B-NTRK1 overexpression significantly increased the proliferation and adhesion-dependent colony formation by activating MAPK and PI3K-AKT signaling pathways. (**A**) PCR was used to verify the expression of KIF5B-NTRK1 arrangement in A549 and H1755 cells. (**B**) Western blotting was used to verify the expression of KIF5B-NTRK1 arrangement in A549 and H1755 cells. (**C**) The effect of KIF5B-NTRK1 on the proliferation of A549 and H1755 cells was analyzed by CCK-8 assay. (**D**) The effect of KIF5B-NTRK1 on the proliferation of A549 and H1755 cells was analyzed by adhesion-dependent colony formation assay. (**E**) The effect of KIF5B-NTRK1 on the invasion ability of A549 and H1755 cells was evaluated by transwell assay. (**F**) Western blotting was used to verify the effect of KIF5B-NTRK1 on the key components of MAPK and PI3K-AKT signaling pathways in A549 and H1755 cells. β-actin was used as a loading control. Image J software was used to quantify the key proteins by grayscale value. (**G**) H1755 cells were treated with entrectinib (500 nM) at different times, and the expression levels of p-AKT, p-ERK, and p-MEK were quantified using WB analysis. The grayscale values of the protein bands were measured using ImageJ software. (**H**) Schematic illustration showing KIF5B-NTRK1 exerting oncogenic properties by activating MAPK and PI3K/AKT signaling pathways. ** *p* < 0.01, *** *p* < 0.001, and **** *p* < 0.0001, versus indicated group. ns, not significant, *p* ≥ 0.05; All data are presented as the means ± SEM.

First, we confirmed the overexpression of KIF5B-NTRK1 in both A549 and H1755 stable expression lines by PCR and WB (Figure 5A,B). The molecular weight of the KIF5B-NTRK1 fusion protein is approximately 130 kD. Our findings reveal that KIF5B-NTRK1 overexpression significantly increased proliferation of the A549 and H1755 cells, as demonstrated by the CCK8 assay (Figure 5C). Furthermore, the adhesion-dependent colony formation assay showed that KIF5B-NTRK1 promoted colony formation in both cell models, with increased colony numbers and sizes than that in the control cells (Figure 5D). However, no significant difference was observed in the number of invasive cells between the two cell models (Figure 5E). Taken together, our findings indicate that KIF5B-NTRK1 overexpression may play a crucial role in the development of lung adenocarcinoma.

### 3.3. KIF5B-NTRK1 Activates the MAPK and PI3K-AKT Signaling Pathways

According to research, the NTRK gene has two major downstream pathways including the MAPK and PI3K-AKT signaling pathways. In order to investigate the regulatory mechanism of KIF5B-NTRK1 in LUAD cells, we conducted an examination of the expression levels of p-AKT, p-ERK, and p-MEK. The results reveal that KIF5B-NTRK1 significantly increased the expression levels of p-AKT, p-ERK, and p-MEK relative to the control group (Figure 5F). Additionally, H1755 cells were treated with entrectinib for 0.5, 1, and 4 h, followed by lysis using lysis buffer to collect total protein. After quantification, each sample was mixed with an equal amount of Laemmli buffer and subsequently loaded onto an SDS-PAGE gel for electrophoresis. The results indicate that the expression levels of p-AKT, p-ERK, and p-MEK in the KIF5B-NTRK1 fusion gene group exhibited a significant decrease over the duration of drug treatment. Conversely, in the control group, the expression levels of p-AKT, p-ERK, and p-MEK demonstrated a slight increase with prolonged entrectinib treatment (Figure 5G). In conclusion, KIF5B-NTRK1 facilitates the proliferation of lung cancer cells via the MAPK and PI3K/AKT signaling pathways, whereas entrectinib effectively inhibits this proliferative activity (Figure 5H).

## 4. Discussion

The *NTRK* gene fusions have been detected in a spectrum of malignant tumors. The fusion partners of *NTRK*, as previously described in NSCLC cases, include the *TPM3*, *MPRIP*, *CD74*, *SQSTM1*, *IRF2BP2*, *MPRIP*, *ETV6*, and *TPR* genes [24,25]. Our team has previously described a newNOTCH2-NTRK1 fusion protein in lung adenocarcinoma [23]. Regardless of the N-terminal partners, the C-terminal region of the *NTRK* fusion gene includes the total tyrosine kinase domain [14]. *KIF5B* belongs to a kinesin family member and has been reported to be fused to *ALK* [26] and *RET* [27], but not to *NTRK*. Here, we report a novel *KIF5B-NTRK1* gene chimera in NSCLC for the first time. The novel *KIF5B-NTRK1* gene consists of *KIF5B* (*exon1–24*) and *NTRK1* (*exon1–24*, which comprises the whole tyrosine kinase domain). The fusion gene partners of *NTRK1* often contain oligomerization domains, such as zinc finger (ZF), coiled-coil (C-C), helix–loop–helix (HLH), Phox and Bem1p interaction domains, RNA recognition motifs (RRMs), and/or WD repeats, which are essential for complete downstream kinase activation [28]. Although the role of partner KIF5B remains to be elucidated, it possesses a beta strand and helix domain and is most likely involved in the activation of downstream pathways.

Additionally, our patient’s tumor harbored several other genetic alterations, including a frameshift mutation in PTEN and copy number amplification of the *MDM2* and *FGF4* genes. PTEN is a well-known tumor suppressor that contributes to the PI3K pathway’s negative feedback loop. PTEN functions upstream of the PI3K-AKT-mTOR signaling axis to regulate cell metabolism, growth, migration, and senescence [29]. Loss of PTEN expression is reported in approximately 5–10% of lung adenocarcinoma cases [30]. The role of PTEN mutation (c.741dup p.P248Tfs*5 of *exon7*) in our case needs to be further validated in future studies. MDM2 overexpression is frequently noted in several tumors [31]. MDM2 negatively regulates the tumor suppressor *TP53* gene [32]. It has been discovered that lung adenocarcinoma patients with MDM2 amplification and protein overexpression have a poor prognosis [33]. However, the available cut-off value for copy number amplification has not been defined so far, which was six in our case and its function was uncertain. The *FGF4* gene, which was initially identified as an oncogene in gastric carcinoma, can induce neoplastic transformation of NIH-3T3 cells [34], as well as epithelial–mesenchymal transition (EMT) by modulating calcium ion channels in lung adenocarcinoma [35]. It has been found that the amplification of FGF4 and FGF19 was five times more frequent in smokers with lung squamous cell carcinoma (LSCC) [36]. However, the frequency and potential role of FGF4 amplification in the pathogenesis of lung adenocarcinoma require further investigation.

The presence of genetic rearrangements in *ALK* is relatively common in NSCLC and can effectively be managed with crizotinib. However, the extent and duration of responses may vary for different *ALK* fusion variants [37]. It remains unclear whether the *NTRK* breakpoint locations differ in terms of response to targeted therapy, necessitating further investigation with additional instances. Entrectinib, the first-generation TRK inhibitor, demonstrated efficacy in patients with NTRK fusion-positive solid tumors. Entrectinib is successful in a variety of malignancies, including NSCLC, salivary gland cancer, thymoma, glioblastoma, and malignant peripheral nerve sheath tumor [38]. In the updated analysis, entrectinib demonstrates efficacy with a high objective response rate (ORR) (64.5%) in patients with NTRK fusion-positive NSCLC and consistently yields durable intracranial responses [15]. However, acquired resistance inevitably occurs with targeted therapy. NTRK1 G595R, NTRK1 G667C, and NTRK3 G623R kinase domain mutations have been reported in colorectal cancer [39] and mammary analog secretory carcinoma (MASC) [40]. Fortunately, the patient reported in our case achieved a long PFS of one year after receiving entrectinib. It is crucial to re-biopsy and perform a large panel gene screening if the tumor enlarges.

## Data Availability

The data that support the findings of this study are available on request from the corresponding author (Gengpeng Lin).
